# Grade Group accuracy is improved by extensive prostate biopsy sampling, but unrelated to prostatectomy specimen sampling or use of immunohistochemistry

**DOI:** 10.3389/pore.2023.1611157

**Published:** 2023-06-21

**Authors:** Kristóf Levente Korpás, Lívia Beke, Dániel Varga, László Bidiga, Gábor Méhes, Sarolta Molnár

**Affiliations:** ^1^ Department of Pathology, Clinical Centre, University of Debrecen, Debrecen, Hungary; ^2^ Department of Urology, Clinical Centre, University of Debrecen, Debrecen, Hungary

**Keywords:** prostate cancer, needle biopsy, prostatectomy, grade group, pathology

## Abstract

Assessing the accurate Grade Group of a prostate needle biopsy specimen is essential for choosing the adequate therapeutic modality for prostate cancer patients. However, it is well-known that biopsy Grade Group tends to up- or downgrade significantly at radical prostatectomy. We aimed to investigate the correlation between accuracy and biopsy core number, performed immunohistochemical staining (IHC) or prostatectomy specimen sampling, with the latest also being correlated with higher detection rates of adverse pathological features, e.g., positive surgical margins, higher pathological stage or presence of perineural invasion (PnI status). The study cohort consisted of 315 consecutive patients diagnosed with prostate adenocarcinoma via transrectal ultrasound-guided needle biopsy who later underwent radical prostatectomy. We grouped and compared patients based on Grade Group accuracy, presence of IHC on biopsy, margin status, pathological stage, and PnI status. Inter-observer reproducibility was also calculated. Statistical analyzes included ANOVA, Tukey’s multiple comparisons *post hoc* test, Chi-squared test, and Fleiss kappa statistics. Undergraded cases harboured a significantly lower number of biopsy cores (*p* < 0.05), than accurately graded cases. Using IHC did not affect grading accuracy significantly, nor did the number of slides from prostatectomy specimens. The mean number of slides was virtually identical when margin status, pathological stage and PnI status of prostatectomy specimens were compared. Inter-observer reproducibility at our institute was calculated as fair (overall kappa = 0.29). Grade Group accuracy is significantly improved by obtaining more cores at biopsy but is unrelated to performed IHC. The extent of sampling prostatectomy specimens, however, did not affect accuracy and failed to significantly improve detection of adverse pathological features.

## Introduction

In 2020, prostate cancer was the second most commonly diagnosed malignancy of males; with the repertoire of screening and treating modalities available today, 375,304 deaths were still reported worldwide [1]. Nowadays, diagnoses are almost exclusively made early, following the revolutionary introduction of prostate-specific antigen screening in the late eighties. Treatment options for localized or locally advanced prostate cancer include hormonal ablation therapy, radiotherapy and radical prostatectomy (RP). Guidelines indicate choosing a treatment modality based on the patient’s risk for disease progression: RP is the gold standard for high-risk patients, while it is most beneficial for low- or very low-risk patients to choose active surveillance. Besides clinical stage, PSA level and comorbidity-adjusted life expectancy, the pathological grade is a primary determinant of patient risk [2]. For the assessment of pathological grade, the Gleason score-based Grade Group system established at the 2014 International Society of Urological Pathology (ISUP) Consensus Conference is used, primarily on needle biopsy specimens [3]. Recent papers suggest that Grade Group (GG) is likely to be a static parameter set early in tumorigenesis, and grade progression is uncommon [4, 5]. However, the question of grade progression has remained controversial [6, 7].

It is beneficial, if not essential, to perform immunohistochemical staining on biopsy samples to ensure the spotting of malignant glands. The most commonly used target antigens are basal cell markers p63 and 34βE12 and neoplastic acinar cell marker α-methylacyl-CoA racemase (AMACR) [8]. For patients undergoing RP, post-RP treatment depends on various clinical and pathological data, including pathological stage, surgical margin status, and final GG.

In the present study, we aimed to see whether an extended number of cores obtained at biopsy, extended RP specimen sampling or performing AMACR/34βE12/p63 IHC on biopsy specimens improves concordance between biopsy and RP GGs. In addition, we hypothesized that extended specimen sampling at pathology results in a higher detection rate of adverse features, e.g., positive surgical margins, perineural invasion, and higher pathological stage (presence of extraprostatic extension or seminal vesicle invasion).

At our institute and in most community settings, biopsy samples are taken by several urologists and evaluated by numerous general pathologists; thus, inter-observer reproducibility frequently biases the accurate grading of biopsy or RP samples. That is why our aims included calculating the inter-observer reproducibility at our institute and identifying morphological pitfalls that could lead to over- or undergrading different prostate samples.

## Materials and methods

### Study settings

In this retrospective study, we analyzed data of men diagnosed with prostatic adenocarcinoma via transrectal ultrasound-guided needle biopsy who later underwent RP at our institute during the period from January 2016 to November 2021. Cases with pre-RP neoadjuvant treatment and more than 1 year between biopsy and RP were excluded. Eventually, a total of 315 consecutive cases met the inclusion criteria. These cases were then grouped based on the concordance between biopsy and RP GGs into accurately graded (*n* = 143), undergraded (*n* = 153) and overgraded (*n* = 19) groups. We compared the number of obtained biopsy cores, presence of IHC on biopsy and multiple factors regarding RP specimens (e.g., slide number, specimen weight, slide/weight ratio, largest tumor diameter) in these three groups. The number of slides was also correlated with adverse pathological features, e.g., presence of positive surgical margin, perineural invasion and higher pT stage. The correction factor for whole-mount slides was 3 (the mean area of tissue embedded in a whole-mount slide equaled three standard slides).

Regarding the RP specimen, the method of processing (total versus partial embedding) was correlated with the weight of the RP specimen and the greatest diameter of the largest tumor nodule. Despite the small number of totally processed cases (*n* = 14), significant differences were found between the two processing methods regarding both parameters. Due to these findings, totally embedded cases were excluded from the statistics regarding specimen sampling, and statistics were done only on partially embedded cases (*n* = 301) to prevent bias of the processing method. As a pre-test analysis to assess whether slide number can be considered an independent variable, it was correlated with the weight of the RP specimen and the greatest diameter of the largest tumor nodule, as one can assume that the extent of the embedding is biased by these two parameters. R^2^ values from both tests indicated no significant correlation with either parameter (Supplementary Figure S1).

### Biopsy procedure

Transrectal ultrasound-guided biopsies were taken using DP-8800 Plus ultrasound machine (Mindray, Shenzhen, Guangdong, China) with an end-fired 7.5 MHz ultrasound probe. The spring-loaded core biopsy gun was equipped with an 18 G needle and notch length of 15 mm. Prior to the procedure, antibiotic prophylaxis with ciprofloxacin was given.

### Pathological processing

Biopsy samples were received in 4% phosphate-buffered formaldehyde solution (cat. no.: HT501640-19L, Sigma-Aldrich Chemie GmbH, Taufkirchen, Germany) mainly in two containers per patient, with cores from each prostate lobe. Cores were transferred into two cassettes and underwent routine formalin-fixed paraffin-embedded processing. 3 μm sections were then made on silanized slides from the selected blocks, stained with hematoxylin and eosin and microscopically evaluated by one of ten general pathologists. Ancillary testing was optional. Histopathological report included the diagnosis, number of cores sent, number of cores containing tumor, GG, estimated percentage of tumor volume in the biopsy specimen, and adverse features, if present. When both lobes were tumorous, the highest GG was reported.

RP specimens were fixed in 4% phosphate-buffered formaldehyde solution overnight, measured and inked. The prostate was sliced in the plane perpendicular to the urethra. Apical and basal (bladder neck) resection margin was then sliced in the sagittal plane and embedded entirely. From this point there was no departmental protocol for further processing of RP specimens, however only 14 RP specimens were totally and 301 partially embedded. Vast majority of partially processed specimens were embedded at least in 50%. Slides were then submitted for further histopathological evaluation. 3 μm sections were hematoxylin and eosin-stained and microscopically evaluated by one of ten general pathologists. Pathological report included size of prostate, the number and location of tissue blocks, tumor focality, localization and size, the estimated percentage of tumor volume in the specimen, histological diagnosis, GG, presence or absence of adverse features (intraductal carcinoma, capsule infiltration, extraprostatic extension and its localization, bladder neck, seminal vesicle, lymphovascular and perineural invasion), surgical margin status and pathological stage.

### Immunohistochemistry

We also analyzed the effect of IHC on GG accuracy. Either single AMACR, 34βE12, p63 staining or a dual or triple cocktail of those was performed. For simplicity purposes, the triple staining protocol is described herein. Routine formalin-fixed paraffin-embedded processing of biopsy samples was done as detailed earlier. Immunohistochemical staining was performed in a BenchMark Ultra immunostaining machine (Roche Diagnostics GmbH, Penzberg, Germany). The deparaffinization of the sections was automated. Antigen retrieval was performed in Cell Conditioning Solution (ULTRA CC1) Tris-based buffer (pH 8.5, cat. no.: 950-224, Roche Diagnostics GmbH, Penzberg, Germany) for 48 min at 100°C. IHC staining started with p63 mouse monoclonal antibody (clone: DAK-p63, cat. no.: M731701-2, Dako, an Agilent Technologies Company, Glostrup, Denmark) and 34βE12 mouse monoclonal antibody (clone: 34βE12, cat. no.: M063001-2, Dako, an Agilent Technologies Company, Glostrup, Denmark) cocktail at a dilution of 1/200 for both p63 and 34βE12. The incubation lasted for 48 min at 37°C. The immunohistochemical reaction was detected with OptiView DAB IHC Detection kit (cat. no.: 760–700, Roche Diagnostics GmbH, Penzberg, Germany). After the first IHC staining, incubation was continued with AMACR mouse monoclonal antibody (clone: 13H4, cat. no.: M361601-2, Dako, an Agilent Technologies Company, Glostrup, Denmark) at a dilution of 1/200. The sections were detected using the ultraView Universal Alkaline Phosphatase Red Detection Kit (cat. no.: 05269814001, Roche Diagnostics GmbH, Penzberg, Germany), and overstaining was done using Hematoxylin II solution (cat. no.: 790–2208, Roche Diagnostics GmbH, Penzberg, Germany) according to the manufacturer’s instructions.

### Inter-observer reproducibility

To assess inter-observer reproducibility, three pathologists evaluated GGs of 20 consecutive prostate biopsies with 12 cores from the study cohort. Only the final given GG of each case was considered. The degree of agreement was defined as *Landis et Koch* described: a kappa value of <0 indicates no agreement, 0–0.20 indicates slight, 0.21–0.40 indicates fair agreement, while a kappa value of 0.41–0.60 means moderate, 0.61–0.80 means substantial, and 0.81–1 means near-perfect agreement [9].

### Visualization

Ten random cases were chosen from the undergraded and overgraded groups and examined thoroughly, looking for possible pitfalls for misgrading. Examples were then photographed using Nikon Eclipse E200 light microscope (Nikon Corporation, Tokyo, Japan), TrueChrome Metrics camera and TCapture 5.1.1 software (Tucsen Photonics Co., Ltd., Fujian, China).

### Statistical analyses

One-way ANOVA with Tukey’s multiple comparisons *post hoc* test were used to calculate the significance of difference among accurately graded, under-, and overgraded groups. To assess the difference between groups of adverse features, unpaired t-test was used. Chi-squared and Fisher’s exact test was the test of choice when comparison of stained and unstained groups. Interobserver reproducibility was calculated using Fleiss kappa. All statistical analyses were performed using GraphPad Prism 9.4.1 (GraphPad Software, Inc., San Diego, CA, United States) and *p* < 0.05 was considered significant.

## Results

The distribution of ISUP GGs is demonstrated in Table 1. The majority of biopsy samples were graded as GG 1 (64.4%), while in the case of RP specimens, the most commonly given GG was 2 (42.9%).

**TABLE 1:** Grade Group distribution among biopsy and radical prostatectomy specimens (n = 315).

		n (*%*)
Biopsy specimens		
	Grade Group 1	203 (*64.4%*)
	Grade Group 2	75 (*23.8%*)
	Grade Group 3	8 (*2.5%*)
	Grade Group 4	24 (*7.6%*)
	Grade Group 5	5 (*1.6%*)
Radical prostatectomy specimens		
	Grade Group 1	91 (*28.9%*)
	Grade Group 2	135 (*42.9%*)
	Grade Group 3	32 (*10.2%*)
	Grade Group 4	33 (*10.5%*)
	Grade Group 5	24 (*7.6%*)

Italic refers to percentage (%) of the total

As presented in Table 2, 45.4% of all cases were graded accurately at biopsy, whereas 48.6% were under-, and 6.0% were overgraded. 81.6% of all cases were graded accurately or within one GG.

**TABLE 2:** Concordance of biopsy and radical prostatectomy Grade Groups in our study (n = 315).

Concordance		n (*%*)
Accurately graded		143 (*45.4%*)
Undergraded total		153 (*48.6%*)
	Undergraded by 1 Grade Group	103 (*32.7%*)
	Undergraded by 2 Grade Groups	22 (*7.0%*)
	Undergraded by 3 Grade Groups	20 (*6.4%*)
	Undergraded by 4 Grade Groups	8 (*2.5%*)
Overgraded total		19 (*6.0%*)
	Overgraded by 1 Grade Group	11 (*3.5%*)
	Overgraded by 2 Grade Groups	7 (*2.2%*)
	Overgraded by 3 Grade Groups	1 (*0.3%*)

Italic refers to percentage (%) of the total

**FIGURE 1:**
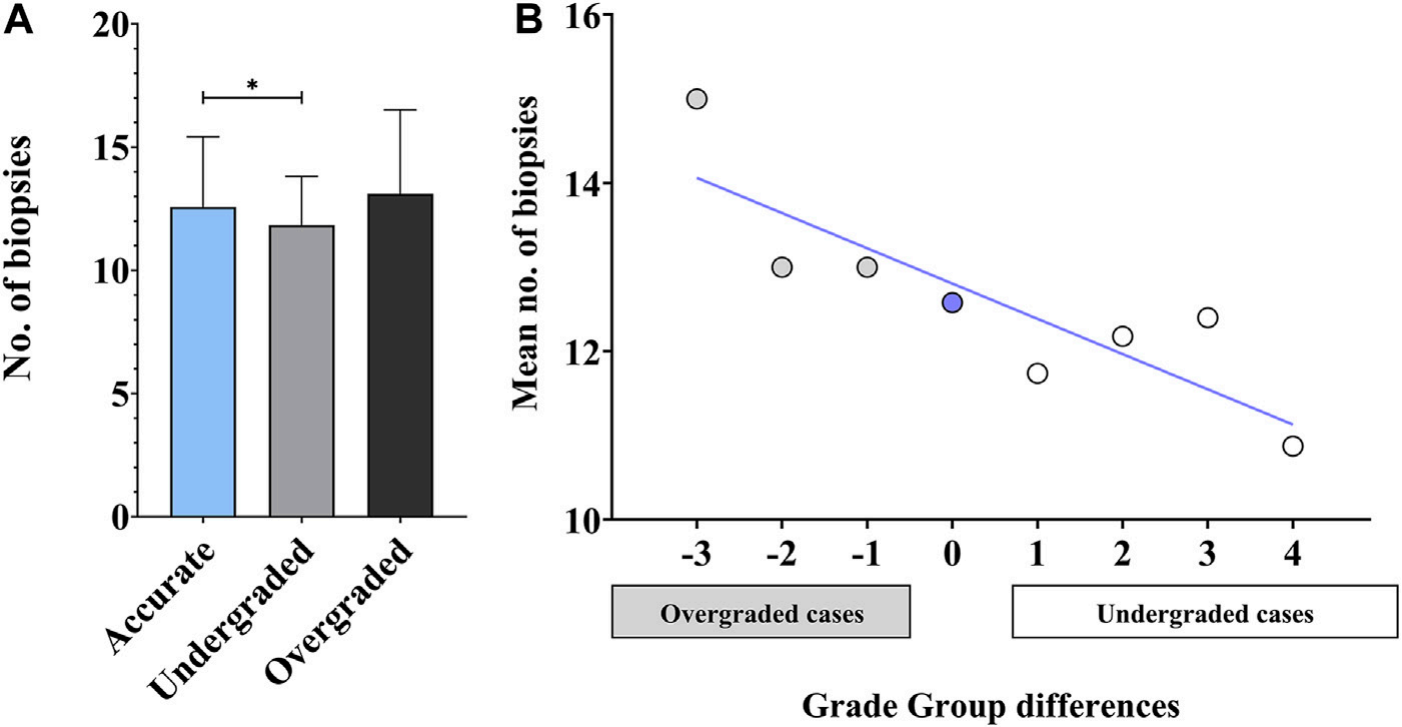
Accuracy of biopsy and radical prostatectomy Grade Groups—biopsy core numbers. **(A)** Statistical comparison of biopsy core numbers in accurately, under-, and overgraded groups. (*n* = 315; mean ± SD; ANOVA, Tukey’s multiple comparisons test; *p < 0.05) **(B)** Mean number of biopsy cores plotted against Grade Group differences (calculated as [*prostatectomy Grade Group*]*—*[*biopsy Grade Group*]) (*n* = 315; blue line: linear fit; R^2^ = 0.74).

The mean number of cores obtained at biopsy was 12.58 ± 2.84, 11.84 ± 1.97 and 13.11 ± 3.41 (range 7–25) in accurately, under-, and overgraded groups, respectively. The difference between these three groups appeared to be significant (*p* = 0.0132). In cases with concordant biopsy and RP GGs, the number of cores obtained at biopsy was significantly higher than in undergraded cases (*p* = 0.0313). The mean number of biopsy cores in the overgraded group was higher than in the other two groups, however, it did not reach statistical significance (Figure 1A). The tendency of overgraded cases harbouring higher and undergraded cases harbouring lower biopsy core numbers than accurately graded cases can be viewed in Figure 1B.

**FIGURE 2:**
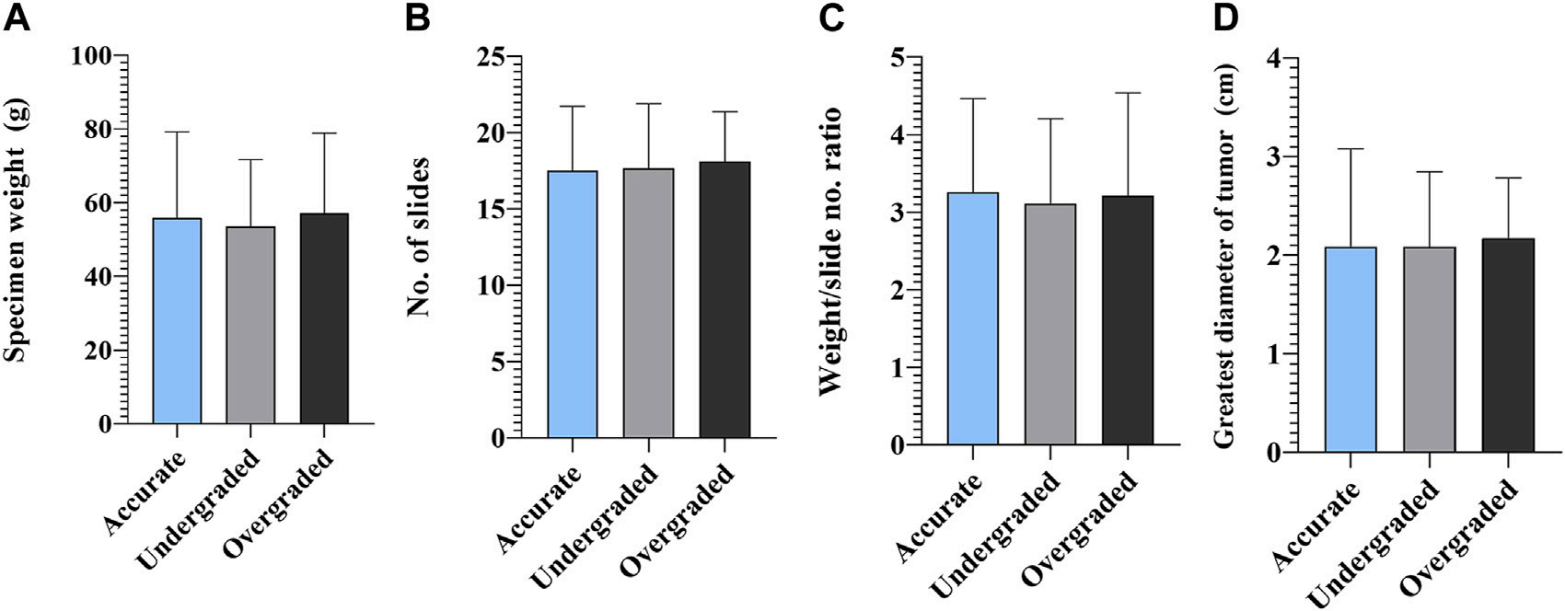
Accuracy of biopsy and radical prostatectomy Grade Groups—radical prostatectomy specimens. **(A)** Statistical comparison of specimen weight, **(B)** number of slides made from prostatectomy specimens, **(C)** specimen weight/slide number ratio and **(D)** greatest diameter of tumor in accurately, under-, and overgraded groups (*n* = 301; mean ± SD).

As far as the extent of RP specimen processing is concerned, pre-test analyzes needed to be run to assess whether total versus partial processing depends on the size of the RP specimen and the greatest diameter of the largest tumor nodule. Totally embedded specimens were significantly less of weight, than those embedded partially (*p* = 0.0094), and the largest diameter of the tumor was significantly smaller (*p* = 0.0424) in the group of totally embedded specimens. Due to these findings, and the relatively small number of cases in the totally embedded group (*n* = 14), we decided to exclude these cases from further statistics and focused only on the group of partially embedded specimens (*n* = 301). The extent of partial RP specimen processing was also correlated with the parameters mentioned above. R^2^ values from both tests indicated no significant correlation with either parameter (Supplementary Figure S1). The mean number of slides was 17.79 ± 4.31, 17.88 ± 4.50 and 18.37 ± 3.37 (range 11–37) in accurately, under-, and overgraded groups, respectively. In terms of accuracy, the number of slides, weight of the specimen and weight/slide ratio indicated no differences between the groups and so did the greatest diameter of the largest tumor nodule (Figure 2).

**FIGURE 3:**
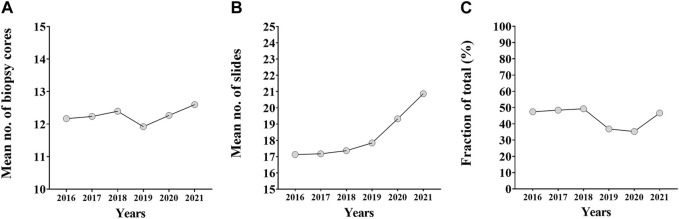
The annual breakdown of biopsy core numbers, prostatectomy specimen slide numbers and fraction of accurately graded cases. **(A)** The annual trend of biopsy core numbers between 2016 and 2021. **(B)** The annual trend of number of slides made from prostatectomy specimens between 2016 and 2021. **(C)** The annual trend of the fraction of accurately graded biopsies between 2016 and 2021.

An annual breakdown of biopsy core and RP slide numbers were also performed. Biopsy core numbers did not change significantly between 2016 and 2021, but had a temporary setback in 2019. In contrast, the number of slides made from prostatectomy specimens showed an increasing tendency between 2016 and 2021 (Figures 3A, B). The annual trend of the fraction of accurately graded biopsies follows the annual trend of biopsy core numbers: the latter reached a nadir in 2019, while the fraction of accurately graded cases had a setback in the same and following year (Figure 3C).

**FIGURE 4:**
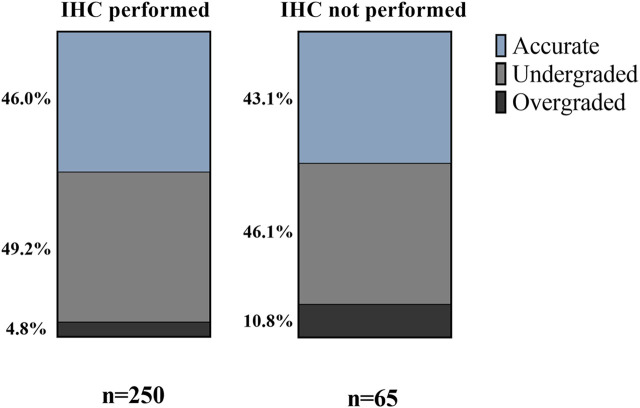
Effect of immunohistochemical staining on Grade Group accuracy—percentages of accurately, under- and overgraded cases in IHC groups.

Using AMACR/p63/34βE12 IHC on biopsy samples did not affect grading accuracy significantly (*p* = 0.1976). We found, however, a slightly larger proportion of accurately graded biopsies when IHC was performed. Another tendency revealed was the diminished ratio of overgraded cases in the IHC-performed group (4.8% vs. 10.8%; *p* = 0.0823) (Figure 4).

Detailed data regarding adverse pathological features of RP specimens in partially embedded group (*n* = 301) are shown in Table 3. The mean number of slides was virtually identical in the case of surgical margin status and pathological stage. Similarly, an extended number of slides did not result in significant difference regarding perineural invasion.

**TABLE 3:** Adverse features evaluated in radical prostatectomy specimens (n = 301).

Feature		n (*%*)	^a^mean ± SD	*p*-value
Margin status				
	Positive	73 (*24.3%*)	17.78 ± 4.315	0.7218	
	Negative	228 (*75.7%*)	17.58 ± 4.127		
^b^Pathological stage				
	pT3	120 (*39.9%*)	17.80 ± 4.428	0.5627	
	pT2	181 (*60.1%*)	17.51 ± 3.990		
Perineural invasion				
	Pn0	36 (*12.0%*)	18.31 ± 4.006	0.3000	
	Pn1	265 (*88.0%*)	17.54 ± 4.188		

SD, standard deviation.

Italic refers to percentage (%) of the total

^a^ Means of the number of slides in each subgroup are shown.

^b^ All cases were of stage pT2 or pT3.

Among the three pathologists involved in this study, consensus on GGs of biopsy samples was reached in 35%, and the overall Fleiss kappa was calculated as 0.29, which indicates only fair agreement. Of cases with perfect agreement among all three pathologists, the majority was GG 1 (4/7; 57%).

**FIGURE 5:**
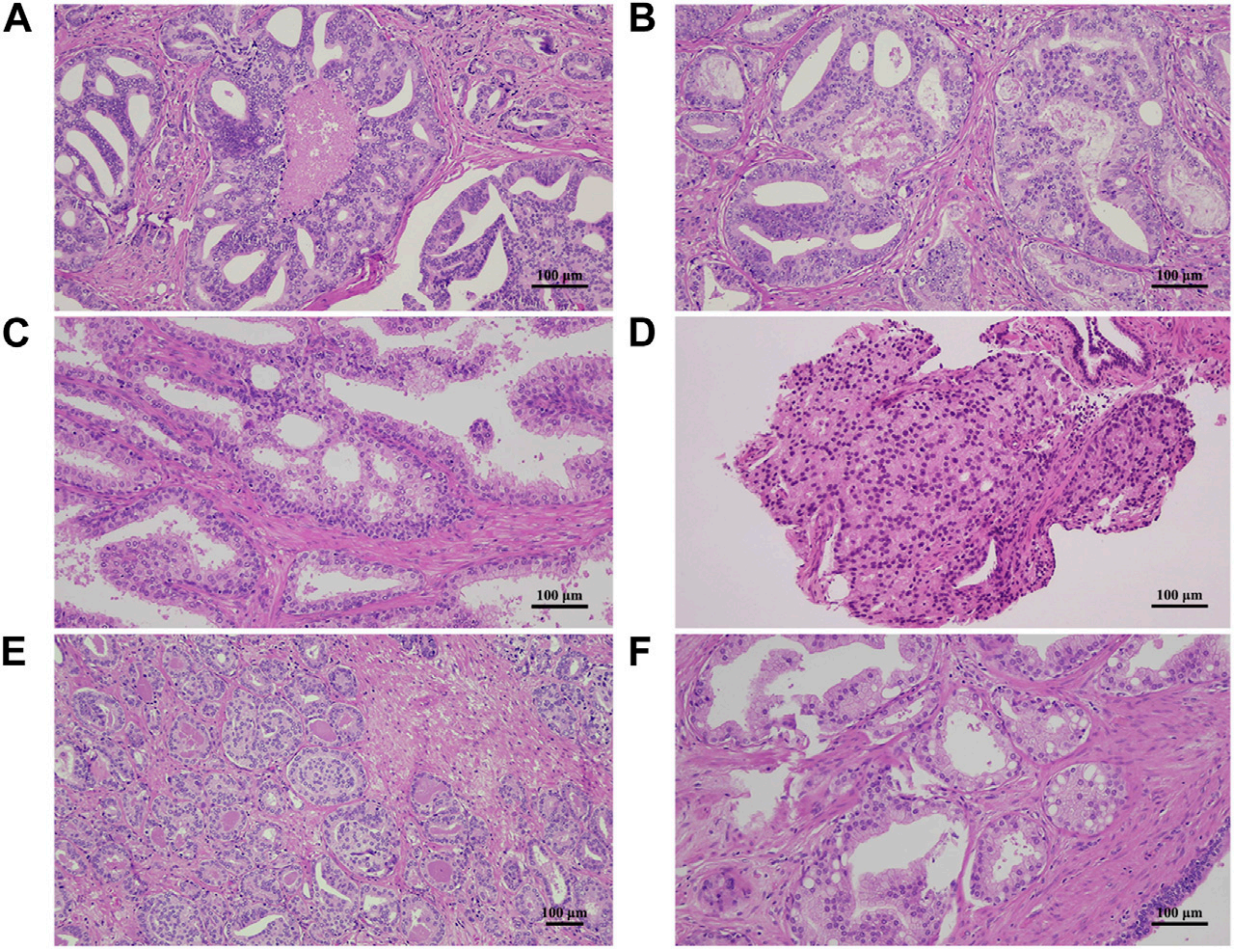
Hematoxylin and eosin-stained sections demonstrating morphologic pitfalls for misgrading prostate adenocarcinoma samples. **(A,B)** Necrotic debris and secretion in a gland with cribriform pattern may be hard to distinguish. **(C)** High-grade prostatic intraepithelial neoplasia may mimic invasive pattern 4 glands, especially with inconsistent basal cell layer and no immunohistochemical stains. **(D)** Closely-packed glands with small lumina can be hard to differentiate from a solid sheet of cells. **(E)** Glomeruloid structures may not be easy to identify when intermixed with pattern 3 glands. **(F)** Cells containing intracellular mucin in a nested or glandular pattern may give the impression of a cribriform gland.

After evaluating 10 randomly chosen samples, we identified the following five morphological patterns that might be misleading when grading prostate needle biopsy or RP specimens:

- Evaluating necrosis within malignant glands with cribriform patterns may be challenging due to the resemblance of necrotic debris to actual glandular secretions. Cribriform pattern with necrosis is Gleason pattern 5, whereas without necrosis, it is Gleason pattern 4 (Figures 5A, B).
- Differentiating between cribriform high-grade prostatic intraepithelial neoplasia and invasive glands with cribriform patterns can be hard, especially when the basal cell layer shows focal integrity deficits. This can lead to invalid reported percentages of pattern 4 glands and may change the final GG (Figure 5C).
- Small lumina may be overlooked in areas with closely packed glands. A solid sheet of cells is Gleason pattern 5, while the presence of abortive glands is Gleason pattern 4 (Figure 5D).
- Gleason pattern 4-equivalent glomeruloid structures are also not easy to identify, especially at low power magnification, when intermixed with Gleason pattern 3 glands (Figure 5E).
- Cohesive cells containing intracellular mucin are frequently encountered in prostate samples. In nested or glandular patterns, they can mimic small cribriform glands. Invasive glands harbouring cribriform pattern equals Gleason pattern 4, whereas non-cribriform structures are considered Gleason pattern 3 or less (Figure 5F).

## Discussion

Accurately grading prostate biopsy samples is essential for choosing the best treatment option for prostate cancer. It saves patients eligible for active surveillance from RP and its side effects and also from undertreatment and progressing into locally advanced or metastatic disease.

Undergrading and overgrading prostate needle biopsies is a well-known phenomenon. A meta-analysis of 16 studies with nearly fifteen thousand patients by Cohen et al. demonstrated an overall accuracy of 63% (range 53%–74%). Undergrading occurred in 30% (range 6%–36%), and overgrading was found in 7% (range 4%–28%) of the cases [10]. In the present study, overall accuracy was found to be 45%, which is markedly inferior to the meta-analysis results; samples were undergraded in 49% and overgraded in 6% of all cases. This may be due to the fact that at our institute—similarly to other minor academic or community centres—mainly general pathologists evaluate and grade prostate samples instead of specified urogenital pathologists. In addition, as Danneman et al. suggested, the newly introduced GG system, in which Gleason score 7 is divided into GG 2 (3 + 4) and 3 (4 + 3), plays a role in poorer accuracy [11].

We found a significant difference in the number of biopsy cores between accurately, under-, and overgraded cases. The group of accurately graded cases harboured a significantly higher mean number of cores than the group of undergraded cases, but interestingly had a lower mean number of cores than the group of overgraded cases. The current prostate biopsy evaluation protocol could be one possible explanation for why overgraded cases harbour higher core numbers: besides the most common Gleason pattern in the sample, the highest-graded pattern also needs to be considered to calculate the GG of each case. When a high number of biopsy cores are taken, the chances of sampling a small focal high-grade pattern are greater. Since the generally poor accuracy of biopsy GG to predict RP GG, several studies have addressed the question of extended biopsy schemes and their effects on concordance. Many of them reported findings consistent with ours [12–15]. However, other studies have failed to show significant improvement in accuracy when using extended biopsy schemes [16, 17]. Miyake et al. grouped cases based on the biopsy core number and found significantly more concordant cases in the group with 10 or more cores, than in the group with less than 10 cores. When subdividing the groups according to initial Gleason scores, this correlation proved to be significant only in specimens with Gleason score of 6 and below (equivalent to GG 1), but the core number did not affect accuracy significantly in cases with higher GGs [18]. A prostate volume-controlled study conducted by Antunes et al. also reported significantly better concordance in the group of 10 or more cores than in groups with 6 or 8 cores, but only in cases of prostates below 50 cm^3^ [19].

Immunohistochemical staining of prostate carcinoma marker AMACR and basal cell markers p63 and 34βE12 are widely-used tools in detecting neoplastic prostate glands lacking basal cell layer. Detection rates have been reported higher when a double or triple cocktail was used, but single staining is also accepted when encountering small foci of suspicious glands, according to the 2014 ISUP recommendations [8, 20–22]. While several studies have addressed the role of IHC in detecting prostate cancer and its mimickers, there are no studies, to date, that have investigated the effects of IHC on the grading accuracy of biopsy samples. Since cribriform pattern equals Gleason pattern 4, noting areas of cribriform high-grade prostatic intraepithelial neoplasia as invasive malignant glands or *vice versa* can lead to over- and underestimating Gleason pattern 4 percentage, resulting in potential under- or overgrading of biopsy samples. In the present study, we failed to demonstrate significantly improved accuracy when IHC was performed. However, a slightly larger proportion of accurately graded cases were found in the group with IHC. A more noticeable finding was the diminished ratio of overgraded cases in the IHC-performed group, which leads to the assumption that not performing IHC is more likely to result in evaluating a suspicious cribriform area rather invasive than non-invasive, thus calculating its pattern into the GG.

In recent decades, multiple studies with numerous approaches have investigated different sampling and processing methods (various partial versus total embedding, standard versus whole-mount slides) for RP specimens. The need for cost-effectiveness and decreased workload at pathology laboratories opposes the need for as little loss of essential information as possible. In most reports, the extent of lost important information, e.g., adverse pathological features, was in primary focus. In a recent paper by Collette et al., the findings of a dozen studies have been summed [23]. Seven of them reported no significant information loss when using partial or alternate slice sampling, thus favouring this RP handling method; [24–30]. The rest promoted total embedding [31–35]. Of those, Desai et al. reported poorer patient outcomes with partial embedding [31]. Inconsistent results from these studies have contributed to the fact that an overall consensus has failed to be made; however, most guidelines recommend total embedding with large-format histology [36, 37]. In our study, specimens were either totally or partially embedded, because there was no departmental protocol for RP specimen handling. However, the former group contained only a small number of cases and the processing type appeared to be biased by the weight of the specimen and the greatest diameter of the largest tumor nodule. Because of these factors we chose not to include totally embedded cases into further statistical analyses and focused only on the partially embedded group. Since the number of slides made from RP specimens acted as an independent variable in our settings, we used this parameter for comparison and found virtually no difference between groups of pT2 and pT3 cases, positive and negative surgical margin cases or Pn0 and Pn1 cases. Furthermore, we found no significant difference in the number of slides in accurately, under- or overgraded groups. These findings suggest that generally when a relatively large specimen and/or tumor is partially embedded (preferably alternating slices), the extent of sampling and processing does not necessarily affect detection rates of adverse pathological features or GG accuracy.

Subjectivity is an intrinsic feature of grading systems, and the Grade Group system is no exception. In centres where numerous general, non-specialized pathologists evaluate specimens of all kinds, inter-observer variability is an inevitable phenomenon that applies to all types of samples. In an optimal case, pathologists work with perfect intra- and inter-observer reproducibility; however, in reality, inter-observer reproducibility of most centres leans towards being moderate. In a publication by Ozkan et al., 407 slides from 34 consecutive cases were evaluated and scored by two general pathologists, who both were trained on the 2005 ISUP Gleason grading system and Epstein’s modification; inter-observer reproducibility was found moderate (overall kappa value of 0.39) [38]. Similarly, another study where 23 general pathologists scored biopsy samples from 37 patients also reported moderate reproducibility of the Gleason grading system (overall kappa = 0.49) [39]. Singh et al. conducted a study in which 20 biopsy/transurethral prostate resection samples were evaluated by 21 general pathologists and showed fair to moderate reproducibility among professionals [40]. Substantial reproducibility (overall kappa = 0.68) was found among six specialised urogenital pathologists, whereas reproducibility among eight general pathologists was moderate (overall kappa = 0.44) in a study by Oyama et al. [41] With a conclusion of tutorials and training being highly beneficial, Mulay et al. showed that reproducibility between four pathologists improved after completing a web-based tutorial session; the original kappa value of 0.459 increased to 0.538 [42]. In the current study, three general pathologists evaluated 20 biopsy samples, and the overall kappa value was 0.29, which indicated only fair agreement.

## Limitations

A final comment should be made on the limitations of the present study. When testing accuracy, only the number of obtained biopsy cores was taken into consideration instead of their summed length. In addition, we corrected the number of slides in cases of whole-mount sectioning, as 1 whole-mount slide equaled 3 standard slides, since most—although not all—pathologists claimed using this ratio when doing large-format histology at our institute. Furthermore, as mentioned earlier, our inter-observer reproducibility in Gleason grading was fair, which may bias the results on improved accuracy. Finally, IHC was not performed uniformly: in some cases, only AMACR or one of the basal cell-specific markers was used, whereas in other cases, a cocktail of two or three of them was applied.

## Conclusion

In conclusion, this study reveals that obtaining more cores (at least 6-6 from both lobes) at prostate needle biopsy of men with high serum PSA levels or lesions suspicious for malignancy results in a better concordance between biopsy and RP GGs. The importance lies in the fact that with accurate grading at biopsy, patients eligible for active surveillance are saved from RP and its side effects, and patients with actual high-grade cancer are saved from potential insufficient treatment and progressing into locally advanced or metastatic disease.

We recommend to use a departmental protocol for handling RP specimens. Small specimens (e.g., below 30 g) should not be processed partially. For higher cost-effectivity, larger specimens could be processed partially, with the acclaimed risk of information being lost, since it is questionably essential for further therapeutic decisions. Alternating slice sampling (with additional sampling of basal and apical slices) can decrease this risk.

Although performing IHC on biopsy cores does not affect GG accuracy, it is well known, that IHC improves detection of prostate cancer. Therefore, we recommend including IHC in the biopsy processing protocols.

## Data Availability

The raw data supporting the conclusion of this article will be made available by the authors, without undue reservation.

## Supplementary material

SUPPLEMENTARY FIGURE S1 Correlations to assess whether slide number is independent of the weight of the specimen and the greatest diameter of the tumor. **(A)** Correlation between number of slides from radical prostatectomy specimens and weight of specimens (*n* = 301; blue line: linear fit; R^2^ is noted). **(B)** Correlation between number of slides from radical prostatectomy specimens and greatest diameter of the tumor (*n* = 185; blue line: linear fit; R^2^ is noted).

## Abbreviations

AMACR, α-methylacyl-CoA racemase; GG, Grade Group; IHC, immunohistochemistry; ISUP, International Society of Urological Pathologists; RP, radical prostatectomy.
