# Supplementary figures and images for: Grade Group accuracy is improved by extensive prostate biopsy sampling, but unrelated to prostatectomy specimen sampling or use of immunohistochemistry
Source: Pathol Oncol Res. 2023 Jun 21;29:1611157. doi: 10.3389/pore.2023.1611157 (PMC10319996; doi:10.3389/pore.2023.1611157)

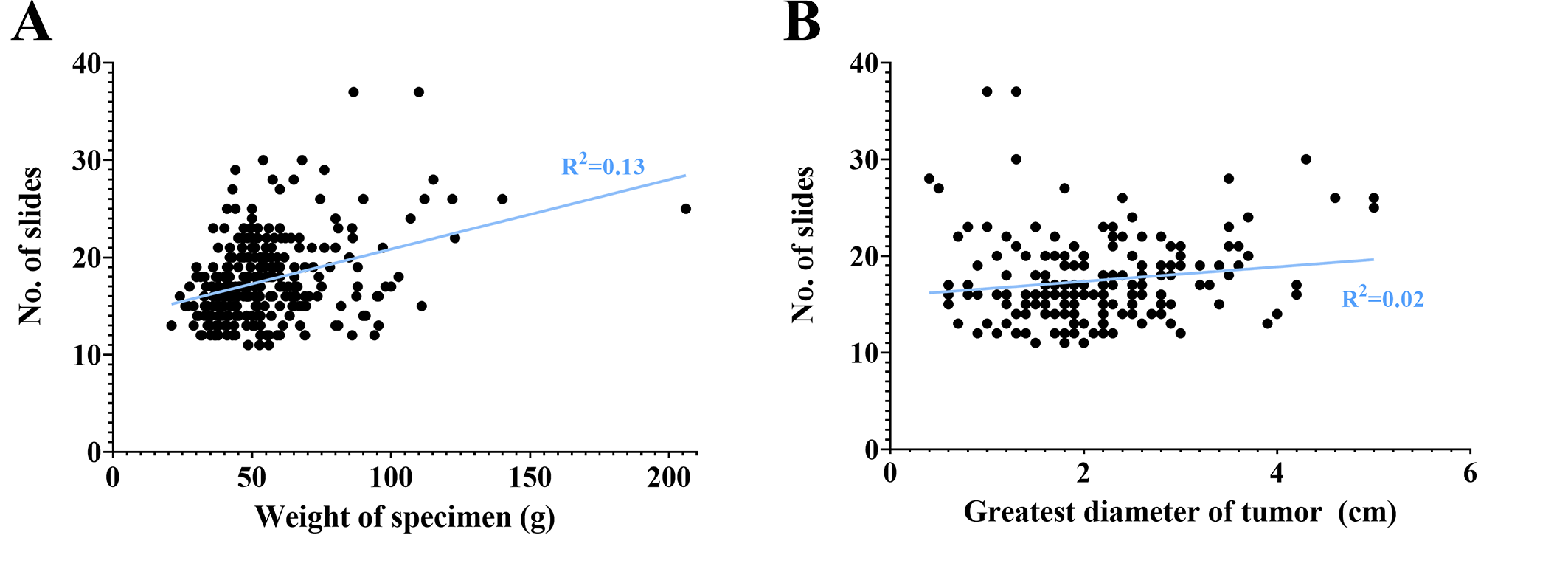

Supplement: Supplementary file 1 [file Image1.TIF]
